# Supplementary material for: Lifestyle mediates the role of nutrient-sensing pathways in cognitive aging: cellular and epidemiological evidence
Source: Commun Biol. 2020 Apr 2;3:157. doi: 10.1038/s42003-020-0844-1 (PMC7118127; doi:10.1038/s42003-020-0844-1)
Supplement: Supplementary file 1 — Supplementary Information [file 42003_2020_844_MOESM1_ESM.pdf]

Supplementary Figures

A

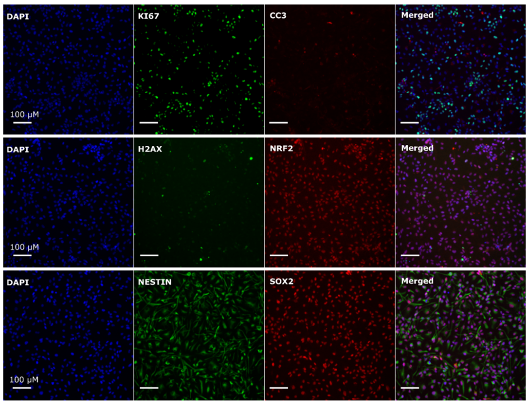

B

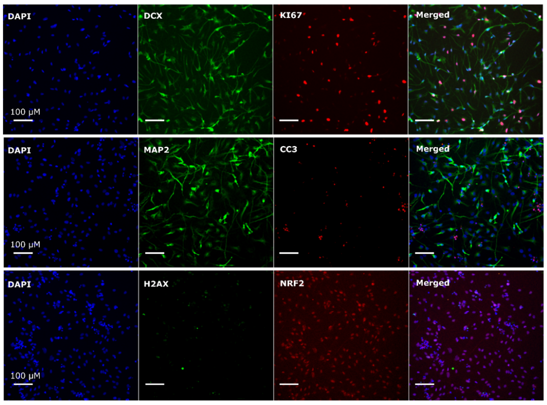

C

Variables included and number of participants	Females : males	MZ : DZ	Min age - Max age Mean (sd)
PAL (no dementia) (2105)	1879:226	1185:906	16.13- 91.82 55.11 ± 14.9
PAL, NART, SNPs (1286)	1209:77	635:651	18.41-91.82 57.22 ± 12.97
PAL, NART, SNPs, nutrition (1165)	1104:61	548:617	18.41-91.82 57.77 ± 12.73
PAL, NART, SNPs, exercise (613)	568:45	321:294	22.52 – 91.82 60.23 ± 12.05

D

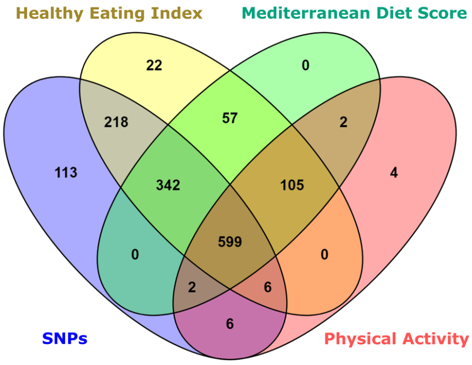

Supplementary Figure 1 Panel of representative images of immunostaining and descriptive statistics of TwinsUK subcohorts. Related to Methods.

Each square represents a field as analysed by the CellInsight software. In each row, the nuclear marker DAPI is in the image followed by **A** Ki67 and CC3 (row 1), H2AX and NRF3 (row 2) or NESTIN and SOX2 (row 3). **B** DCX and Ki67 (row 1), MAP2 and CC3 (row 2) or H2AX and NRF3 (row 3). The final image in each row is the merged image showing all three stains combined. Scale bar representing 100μM is reported in the bottom left corner of each image. **C** Table showing the female to male ratio, monozygotic (MZ) to dizygotic (DZ) ratio and minimum, maximum and mean ± SD age for each of the sub cohorts. **D** Venn diagram showing the overlap between the 4 subgroups described in C.

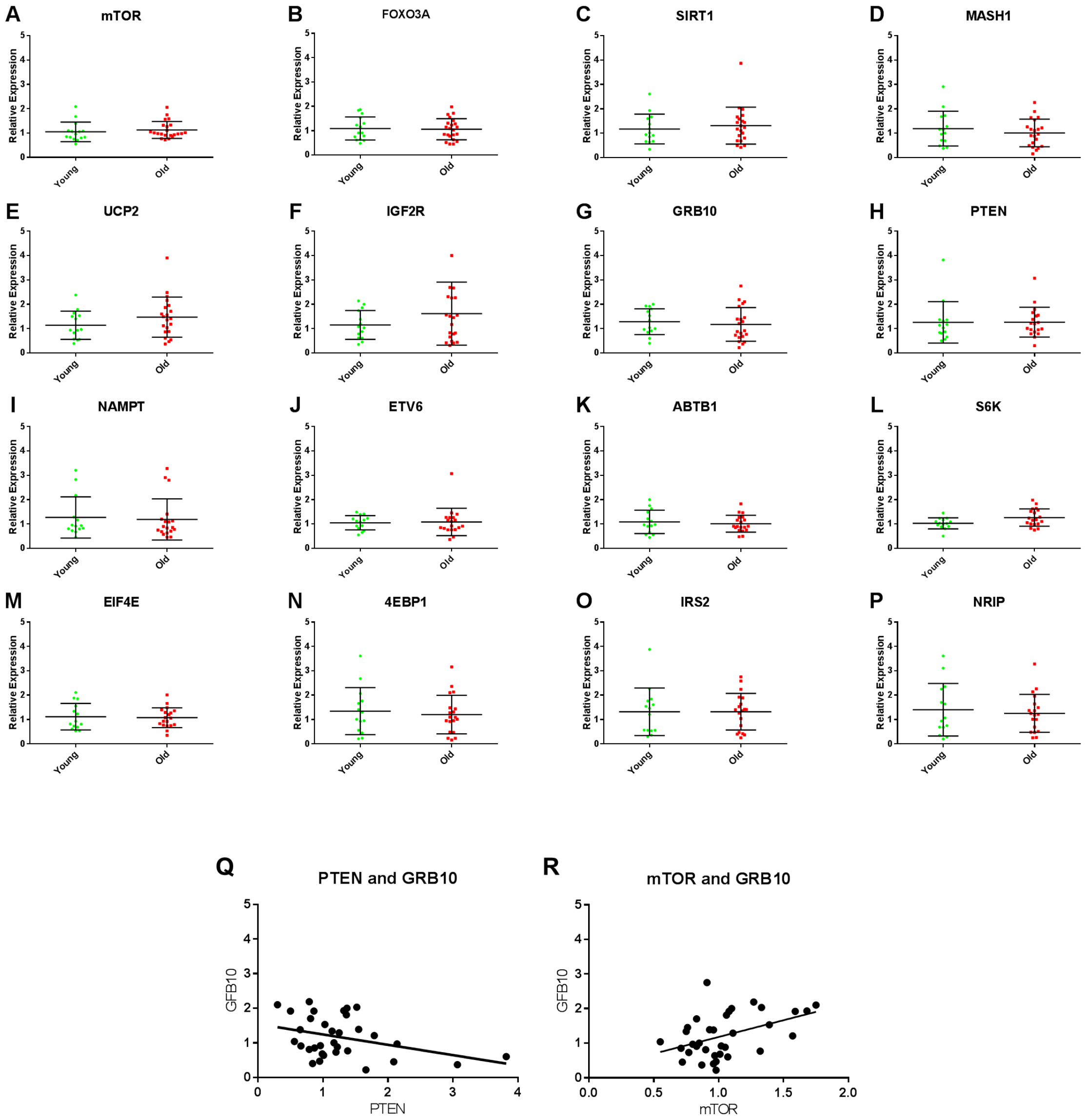
A mTOR
B FOXO3A
C SIRT1
D MASH1
E UCP2
F IGF2R
G GRB10
H PTEN
I NAMPT
J ETV6
K ABTB1
L S6K
M EIF4E
N 4EBP1
O IRS2
P NRIP
Relative Expression
Young
Old
Q PTEN and GRB10
GFB10
PTEN
R mTOR and GRB10
GFB10
mTOR

Supplementary Figure 2 - Gene expression levels of candidate genes remains unaltered following *in vitro* **parabiosis. Related to Figure 2.**

A-P Gene expression levels of the 16 candidate genes in the cells following treatment with serum from young (green) or old (red) serum donors. In all graphs, relative expression (shown as expression relative to average expression by cells treated with young serum) is on the y-axis. Error bars represent standard deviation.

QR Scatterplots and line of best fit showing the association between expression levels of genes known to regulate each other's function or expression. Each dot represents the average expression levels of each gene following treatment of cells with a single serum sample. Correlation analysis was used to check for significant associations. PTEN and GRB10 expression levels were negatively correlated ($p = 0.037$, $r = -0.36$). mTOR and GRB10 expression levels instead, showed positive correlations ($p= 0.007$, $r= 0.44$).

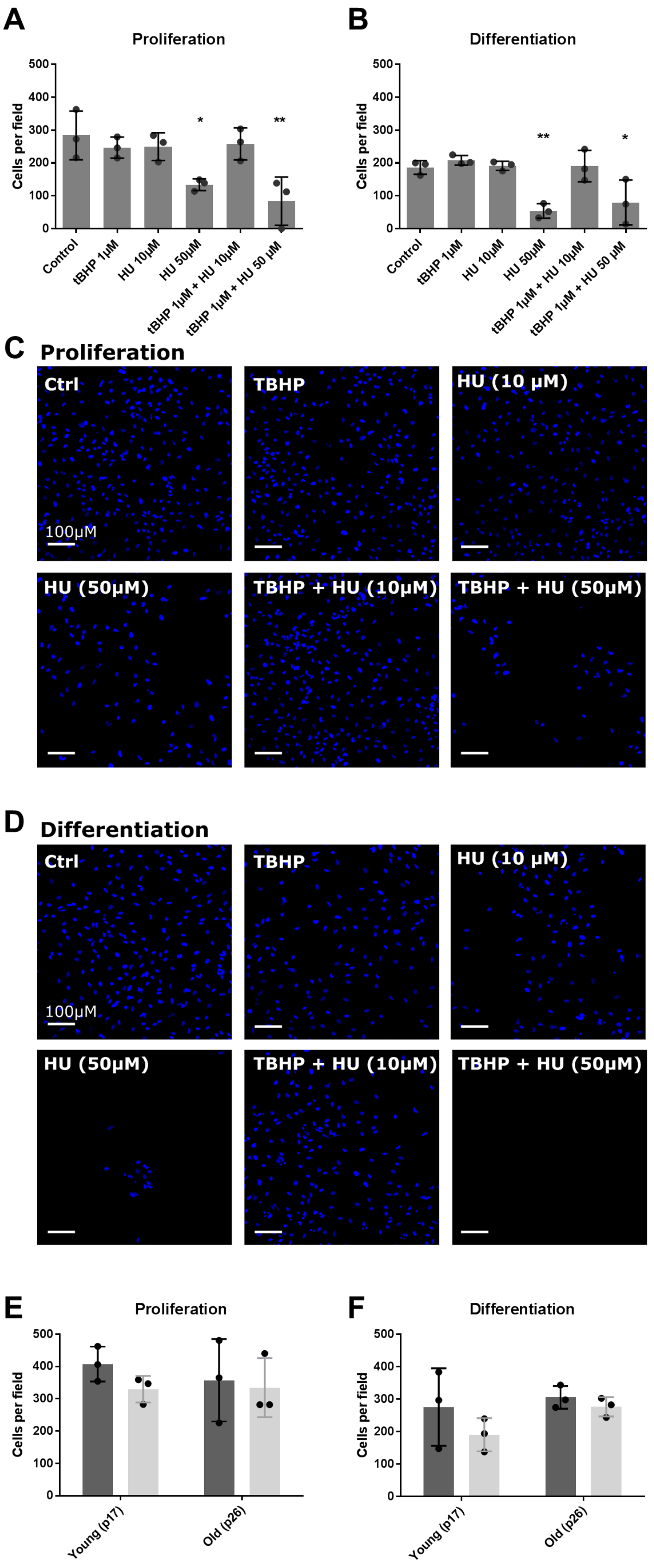

A
Proliferation
Cells per field
500
400
300
200
100
0
*
**
Control
tBHP 1μM
HU 10μM
HU 50μM
tBHP 1μM + HU 10μM
tBHP 1μM + HU 50 μM
B
Differentiation
Cells per field
**
*
Control
tBHP 1μM
HU 10μM
HU 50μM
tBHP 1μM + HU 10μM
tBHP 1μM + HU 50 μM
C
Proliferation
Ctrl
TBHP
HU (10 μM)
100μM
HU (50μM)
TBHP + HU (10μM)
TBHP + HU (50μM)
D
Differentiation
Ctrl
TBHP
HU (10 μM)
100μM
HU (50μM)
TBHP + HU (10μM)
TBHP + HU (50μM)
E
Proliferation
Cells per field
Young (p17)
Old (p26)
F
Differentiation
Cells per field
Young (p17)
Old (p26)

Supplementary Figure 3 – Selecting viable treatment concertation and passage number. Related to Figure 3.

AB Cell number (cells per field) following individual and combination treatments with tert-Butyl hydroperoxide (tBHP) and hydroxyurea (HU). **A** Proliferation assay results, passage 17 cells were seeded and treated after 24 hours with either 1µM tBHP, 10µM HU, 50µM HU, 1µM tBHP + 10µM HU or 1µM tBHP + 50µM HU and incubated for 48 hours. The 50µM HU treatment group showed a 52.9% reduction in cell number relative to the media-only control while the 1µM tBHP + 50µM HU showed a 70.6% reduction in cell number relative to media-only control. Other treatments showed no significant effects on cell number. **B** Differentiation assay results, cells were seeded and treated after 24 hours with either 1µM tBHP, 10µM HU, 50µM HU, 1µM tBHP + 10µM HU or 1µM tBHP + 50µM HU. Cells were incubated with the corresponding treatment for 48 hours, cells were then allowed to differentiate and were incubated with the corresponding treatment for 7 further days. The 50µM HU treatment group showed a 71.1% reduction in cell number relative to the media-only control while the 1µM tBHP + 50µM HU showed a 57.3% reduction in cell number relative to media-only control. Other treatments showed no significant effects on cell number.

CD Representative images of DAPI stain following the proliferation and differentiation assay with tert-Butyl hydroperoxide (tBHP) or hydroxyurea (HU) treatments.

Each square represents a field as analysed by the CellInsight software. DAPI (in blue) stains the cell nucleus by binding to DNA. DAPI stain was quantified using the CellInsight software to quantify cell density. Treatment concentration is reported in the top left corner of each image. **A** DAPI stain following the proliferation assay; 48-hour incubation with treatment. **B** DAPI stain following the differentiation assay; this included a 48-hour incubation during proliferation and a 7-day incubation during differentiation

EF Cell number following proliferation and differentiation assays on treated and untreated passage 17 (p17) and passage 26 (p26) cells. **E** Proliferation assay results, cells at different passage numbers were seeded and incubated with media-only or treated after 24 hours and incubated with 1µM tert-Butyl hydroperoxide (tBHP) + 10µM hydroxyurea (HU) for 48 hours. **F** Differentiation assay results, cells at different passage numbers were incubated in either media only conditions or with 1µM tert-Butyl hydroperoxide (tBHP) + 10µM hydroxyurea (HU) for 48 hours during the proliferation stage and for 7 days during the differentiation stage. Both graphs show no significant changes in cell number due to passage number. Error bars represent standard deviation. One-way ANOVAs followed by Bonferroni post-hoc analysis. 3 biologically indipendent experiments and 3 technical replicates. Significance displayed as ***** $p < 0.05$, ****** $p < 0.01$.

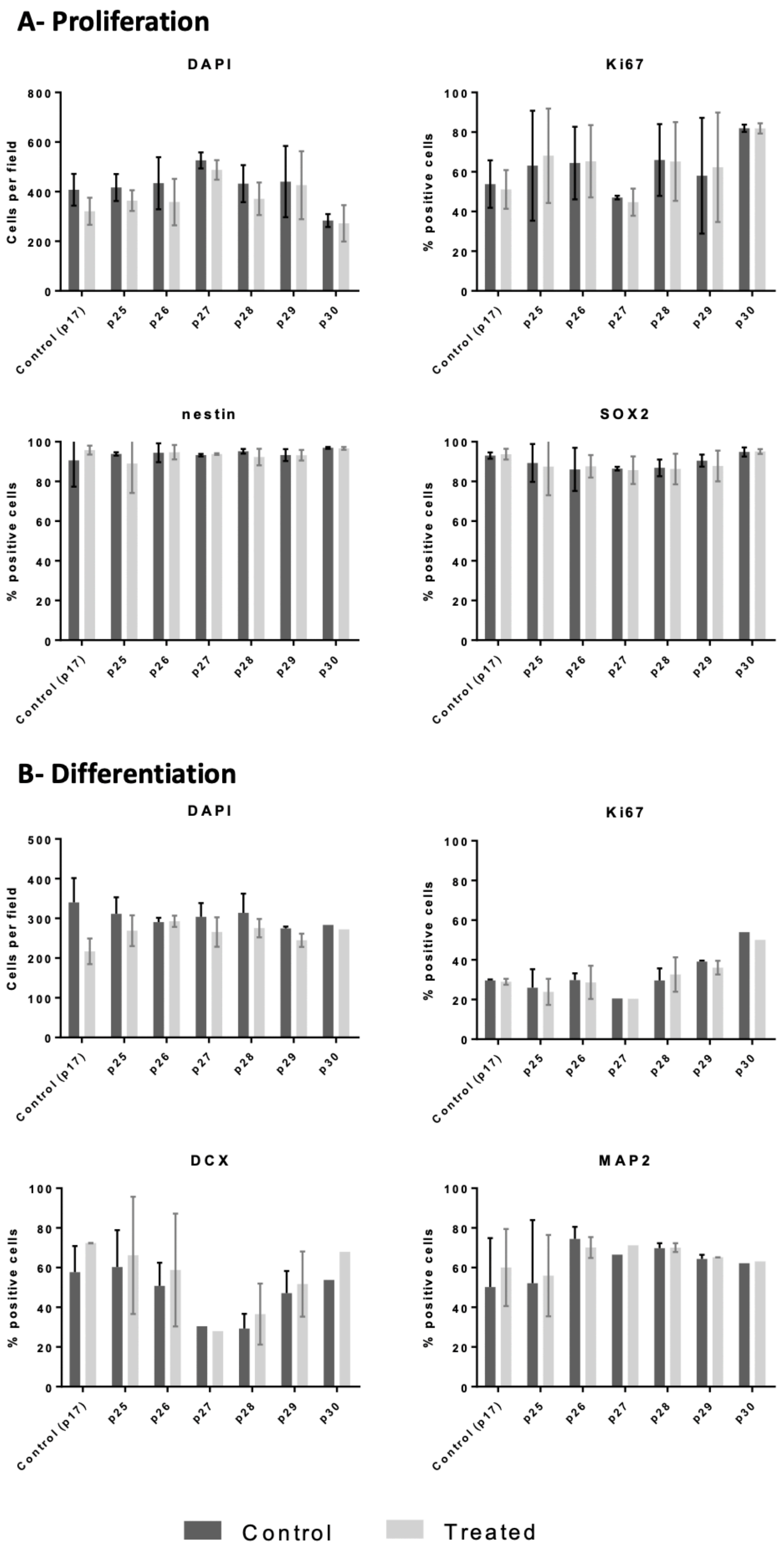
A- Proliferation
DAPI
Cells per field
0
200
400
600
800
Ki67
% positive cells
0
20
40
60
80
100
nestin
SOX2
Control (p17)
p25
p26
p27
p28
p29
p30
B- Differentiation
DAPI
0
100
200
300
400
500
Ki67
DCX
MAP2
Control
Treated

Supplementary Figure 4 – Cells with high passages show no significant variations in the expression of proliferation and differentiation markers.

HPC0A07/03A cells at different passage numbers were seeded and incubated with media-only or treated after 24 hours and incubated with 1µM tert-Butyl hydroperoxide (tBHP) + 10µM hydroxyurea (HU) for 48 hours. Passage numbers included 17 (p17) as a control and several higher passages ranging from passage 25 (p25) to passage 30 (p30) to test whether high passage number cells retained the expression of proliferation and differentiation markers and whether the marker expression varied across different high passages. DAPI was used to quantify the number of cells per field (y-axis); treatment (light grey) appeared to cause slight reductions in cell number at most passage numbers. Besides some fluctuations passage number did not seem to affect cell number.

A- Proliferation - Ki67: Ki67 was used to assess proliferation, the percentage of cells positive for this marker is displayed on the y-axis. Ki67 was expressed at all passage numbers and did not seem affected by treatment (light grey). Nestin and SOX2 graphs show the percentage positive cells (y-axis) for these stemness markers. Cells at all passage numbers retained their stemness as evidenced by largely unchanged SOX2 and nestin expression. In addition, the highest passage cells (p30) showed no obvious difference in marker expression compared to the lowest of the high passage cells (p25).

B- Differentiation - Ki67: Ki67 was used to assess any remaining proliferation during the differentiation assay, the percentage of cells positive for this marker is displayed on the y-axis. Low levels of Ki67 was expressed at all passage numbers with a possible increase at higher passages. It did not seem affected by treatment (light grey). DCX and MAP2 graphs show the percentage positive cells (y-axis) for the neuroblast maker (DCX) and immature neuron (MAP2). Cells retained their ability to differentiate towards a neuronal lineage at high passage numbers and did not appear affected by treatment (light grey). As for the proliferation markers, the highest passage cells (p30) showed no obvious difference in marker expression compared to the lowest of the high passage cells (p25).

Dark grey: media-only controls. Light grey: with 1µM tBHP + 10µM HU. Two biologically independent experiments, 3 technical replicates. Error bars display standard deviation.

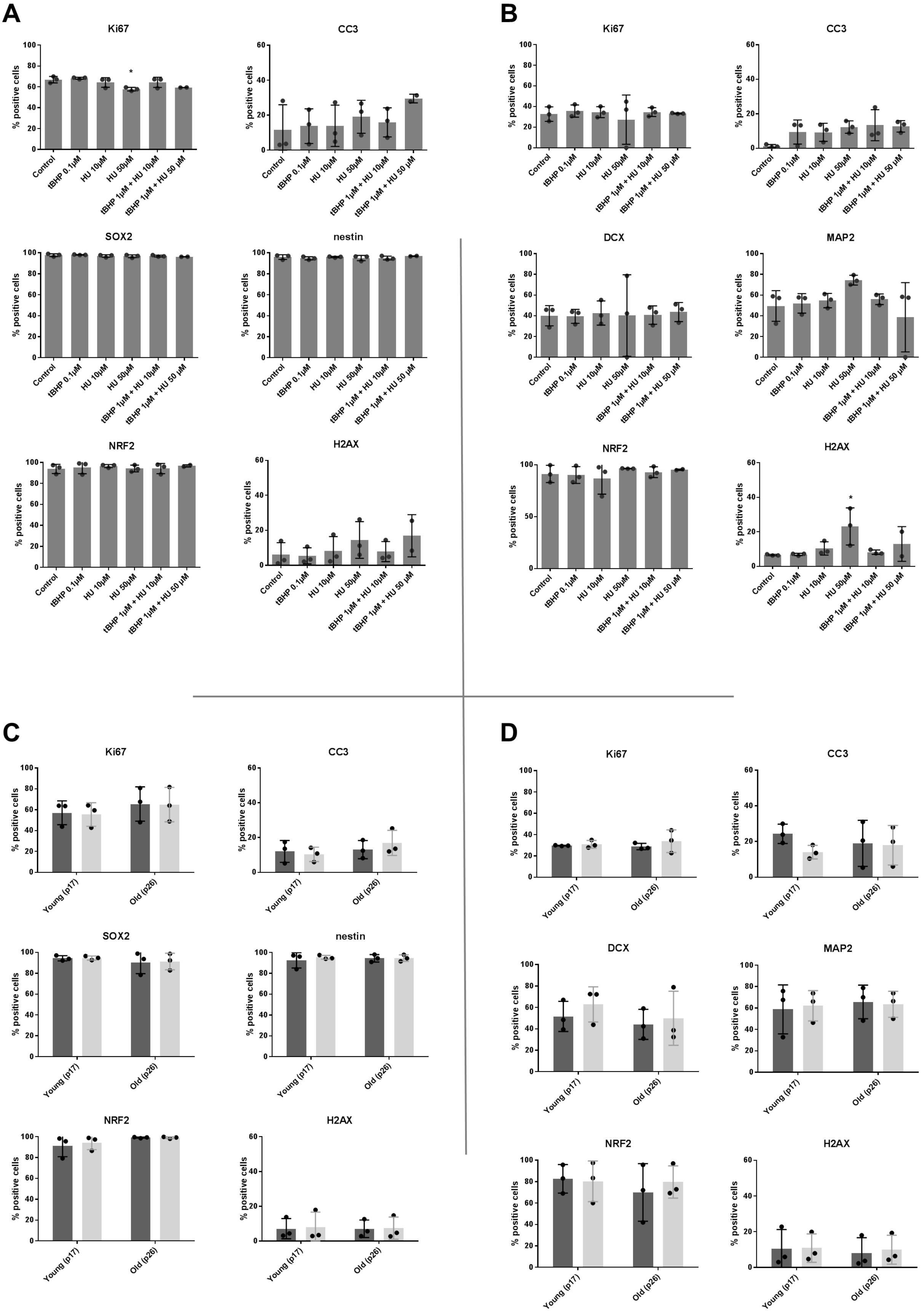
A
B
C
D
Ki67
CC3
SOX2
nestin
NRF2
H2AX
DCX
MAP2
% positive cells
Control
tBHP 0.1μM
HU 10μM
HU 50μM
tBHP 1μM + HU 10μM
tBHP 1μM + HU 50 μM
Young (p17)
Old (p26)
*

Supplementary Figure 5 – No variation in cellular markers of stem cell maintenance following treatment and increased passage number at non-toxic concentrations. Related to Figure 3.

A Proliferation assay results; passage 17 cells were seeded and after 24hrs incubated with either 1µM tert-Butyl hydroperoxide (tBHP), 10µM hydroxyurea (HU), 50µM HU, 1µM tBHP + 10µM HU or 1µM tBHP + 50µM HU for 48 hours. There was a significant reduction in Ki67% positive cells following the proliferation assay in the 50µM HU treatment group

B Differentiation assay results; passage 17 cells were incubated in either media only conditions or with either 1µM tBHP, 10µM HU, 50µM HU, 1µM tBHP + 10µM HU or 1µM tBHP + 50µM HU during the proliferation stage and for 7 days during the differentiation stage. H2AX% positive cells remained largely unchanged besides a significant increase in the 50µM HU treatment group.

C Proliferation assay results; passage17 (p17) or passage 26 (p26) cells were incubated in either media only conditions or with 1µM tBHP + 10µM HU during the proliferation assay.

D Differentiation assay results; p17 or p26 cells were incubated in either media only conditions or with, 1µM tBHP + 10µM HU during the proliferation stage and for 7 days during the differentiation stage.

In each graph the percentage of positive cells for each marker is displayed on the y-axis. One-way (**AB**) or Two-way (**CD**) ANOVAs followed by Bonferroni post-hoc. 3 biologically independent experiments, 3 technical replicates. Error bars display standard deviation. $^*p < 0.05$.

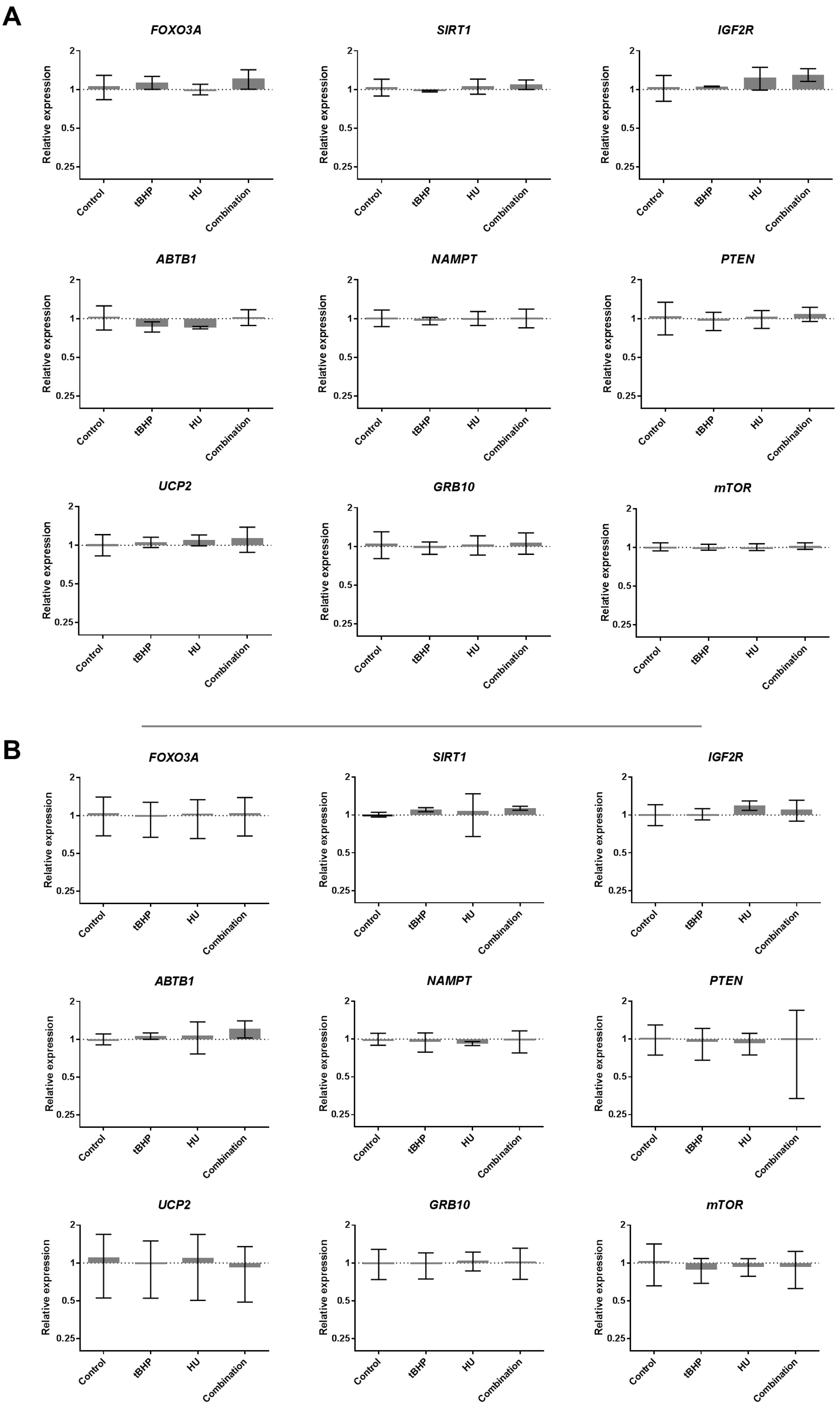
A
FOXO3A
SIRT1
IGF2R
ABTB1
NAMPT
PTEN
UCP2
GRB10
mTOR
Relative expression
2
1
0.5
0.25
Control
tBHP
HU
Combination
B
FOXO3A
SIRT1
IGF2R
ABTB1
NAMPT
PTEN
UCP2
GRB10
mTOR

Supplementary Figure 6 – No variation in candidate gene expression following treatment. Related to Figure 3.

A Proliferation assay results: Passage 17 cells were incubated in either media only conditions (control) or with 1µM tert-Butyl hydroperoxide (tBHP), 10µM hydroxyurea (HU) or 1µM tBHP + 10µM HU (Combination) during the proliferation assay. Y-axis show the expression of each gene relative to control as calculated by the Pfaffl method. FOXO3A, SIRT1, IGF2R, ABTB1, NAMPT, PTEN, UCP2, GRB10 and mTOR expression were not found to be altered following treatment.

B Differentiation assay results: Passage 17 cells were incubated in either media only conditions (control) or with 1µM tert-Butyl hydroperoxide (tBHP), 10µM hydroxyurea (HU) or 1µM tBHP + 10µM HU (Combination) during the proliferation stage and for 7 days during the differentiation stage. Y-axis show the expression of each gene relative to control as calculated by the Pfaffl method.

One-way ANOVAs 3 biologically independent experiments, 3 technical replicates. Error bars represent standard deviation. Y-axis is a log scale.

A

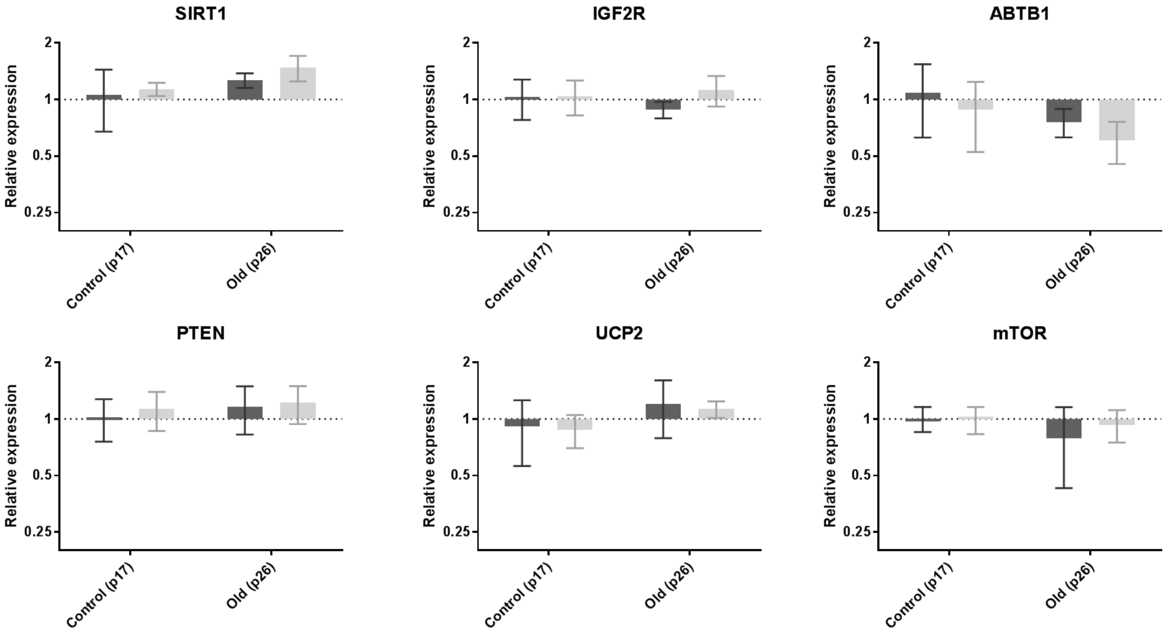
SIRT1
IGF2R
ABTB1
PTEN
UCP2
mTOR
Relative expression
2
1
0.5
0.25
Control (p17)
Old (p26)

B

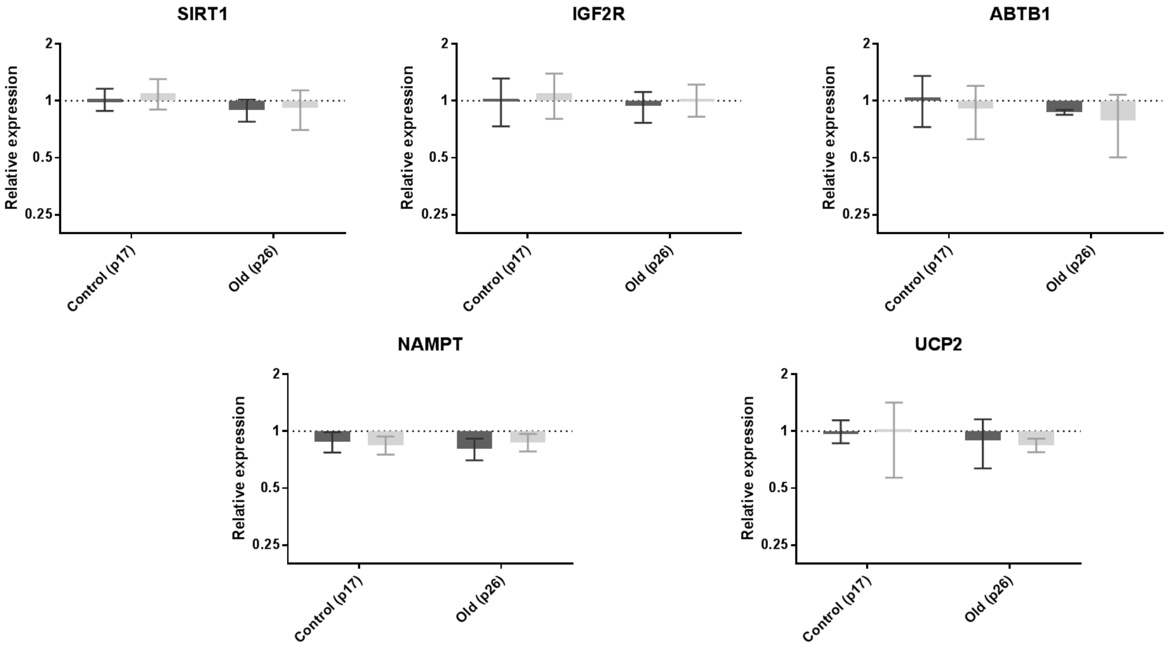
SIRT1
IGF2R
ABTB1
NAMPT
UCP2
Relative expression
2
1
0.5
0.25
Control (p17)
Old (p26)

Supplementary Figure 7 – No variation in candidate gene expression following treatment. Related to Figure 3 C-I.

A Non-significant proliferation assay results: Passage 17 (p17) or Passage 26 (p26) cells were incubated in either media only conditions (control) or with 1µM tBHP + 10µM HU (Treated) during the proliferation assay. Quantitative polymerase chain reaction (qPCR) was used to measure gene expression. Y-axis show the expression of each gene relative to control as calculated by the Pfaffl method.

B Non-significant differentiation assay results: Passage 17 (p17) or Passage 26 (p26) cells were incubated in either media only conditions (control) or with 1µM tBHP + 10µM HU (Treated) during the proliferation assay. Y-axis show the expression of each gene relative to control as calculated by the Pfaffl method.

Two-way ANOVAs, 3 biologically independent experiments, 3 technical replicates. Error bars represent standard deviation. Y-axis is a log scale.
