# Supplementary material for: Lifestyle mediates the role of nutrient-sensing pathways in cognitive aging: cellular and epidemiological evidence
Source: Commun Biol. 2020 Apr 2;3:157. doi: 10.1038/s42003-020-0844-1 (PMC7118127; doi:10.1038/s42003-020-0844-1)
Supplement: Supplementary file 2 — Description of Additional Supplementary Files [file 42003_2020_844_MOESM2_ESM.pdf]

Supplementary Data 1– Excel table showing resulting tag SNPs following pruning.

Each raw SNPs (column A) along with its position (column B and C) and the minor or major allele (column D and E) is reported. SNPs selected as a tag SNPs are indicated by “1” in column F and the best tag SNP for each SNP is reported in column G. The degree of linkage disequilibrium between the SNP and its best tag SNP, measured by r^2, is reported in column H. Best SNPs with an $r^2 > 0.8$ were selected as tag SNPs.

Supplementary Data 2– Excel table showing GEE models results testing the association between each of the 482 SNPs to PAL performance.

Each row reports GEE results of the association between a SNP and PAL performance. SNP, Beta coefficient, Lower confidence interval, Upper confidence interval, p value, number of participants and q value following Benjamini Hochberg correction are reported.

Supplementary Data 3– Excel table showing GEE models results testing the interaction between physical activity (IPAQ) and each of the 482 SNPs on PAL performance.

Each row reports interaction results of each GEE model testing for the interaction between IPAQ and each of the 482 SNPs on PAL performance. SNP, Beta coefficient, Lower confidence interval, Upper confidence interval, number of participants, interaction p value and interaction q value following Benjamini Hochberg correction are reported.

Supplementary Data 4– Excel table showing GEE models results testing the interaction between Mediterranean Diet Score (MDS) and each of the 482 SNPs on PAL performance.

Each row reports interaction results of each GEE model testing for the interaction between MDS and each of the 482 SNPs on PAL performance. SNP, Beta coefficient, Lower confidence interval, Upper confidence interval, number of participants, interaction p value and interaction q value following Benjamini Hochberg correction are reported.

Supplementary Data 5– Excel table reporting the raw data behind graphs shown in Figure 2.

Data from each graph is reported in a separate tab. X and Y axis labels are reported in the first row in each tab.

Supplementary Data 6– Excel table reporting the raw data behind graphs shown in Figure 3.

Data from each graph is reported in a separate tab. X and Y axis labels are reported in the first row in each tab.
