# Supplementary material for: Lifestyle mediates the role of nutrient-sensing pathways in cognitive aging: cellular and epidemiological evidence
Source: Commun Biol. 2020 Apr 2;3:157. doi: 10.1038/s42003-020-0844-1 (PMC7118127; doi:10.1038/s42003-020-0844-1)
Supplement: Supplementary file 9 — Reporting Summary [file 42003_2020_844_MOESM9_ESM.pdf]

nature research

Corresponding author(s): Sandrine Thuret

Last updated by author(s): Oct 4, 2019

Reporting Summary

Nature Research wishes to improve the reproducibility of the work that we publish. This form provides structure for consistency and transparency in reporting. For further information on Nature Research policies, see Authors & Referees and the Editorial Policy Checklist.

Statistics

For all statistical analyses, confirm that the following items are present in the figure legend, table legend, main text, or Methods section.

n/a	Confirmed	
☐	☒	The exact sample size (*n*) for each experimental group/condition, given as a discrete number and unit of measurement
☐	☒	A statement on whether measurements were taken from distinct samples or whether the same sample was measured repeatedly
☐	☒	The statistical test(s) used AND whether they are one- or two-sided<br>*Only common tests should be described solely by name; describe more complex techniques in the Methods section.*
☐	☒	A description of all covariates tested
☐	☒	A description of any assumptions or corrections, such as tests of normality and adjustment for multiple comparisons
☐	☒	A full description of the statistical parameters including central tendency (e.g. means) or other basic estimates (e.g. regression coefficient) AND variation (e.g. standard deviation) or associated estimates of uncertainty (e.g. confidence intervals)
☐	☒	For null hypothesis testing, the test statistic (e.g. *F*, *t*, *r*) with confidence intervals, effect sizes, degrees of freedom and *P* value noted<br>*Give P values as exact values whenever suitable.*
☒	☐	For Bayesian analysis, information on the choice of priors and Markov chain Monte Carlo settings
☐	☒	For hierarchical and complex designs, identification of the appropriate level for tests and full reporting of outcomes
☐	☒	Estimates of effect sizes (e.g. Cohen's *d*, Pearson's *r*), indicating how they were calculated

Our web collection on statistics for biologists contains articles on many of the points above.

Software and code

Policy information about availability of computer code

Data collection	For the epidemiological study, phenotypic and genotypic data were collected and accessed through TwinsUK. See details here: https://twinsuk.ac.uk/resources-for-researchers/our-data/<br>Cellular phenotyping data were collected via CellInsight CX7 High-Content Screening (HCS) Platform
Data analysis	All statistical analyses were performed in R Studio (see methods for packages used) and Prism 6.

For manuscripts utilizing custom algorithms or software that are central to the research but not yet described in published literature, software must be made available to editors/reviewers. We strongly encourage code deposition in a community repository (e.g. GitHub). See the Nature Research guidelines for submitting code & software for further information.

Data

Policy information about availability of data

All manuscripts must include a data availability statement. This statement should provide the following information, where applicable:

- Accession codes, unique identifiers, or web links for publicly available datasets
- A list of figures that have associated raw data
- A description of any restrictions on data availability

Access to the epidemiological data is available and can be requested through the TwinsUK cohort. http://twinsuk.ac.uk/resources-for-researchers/access-our-data/

Raw data from the cellular work can be obtained from the corresponding author.

Field-specific reporting

Please select the one below that is the best fit for your research. If you are not sure, read the appropriate sections before making your selection.

☒ Life sciences ☐ Behavioural & social sciences ☐ Ecological, evolutionary & environmental sciences

For a reference copy of the document with all sections, see nature.com/documents/nr-reporting-summary-flat.pdf

Life sciences study design

All studies must disclose on these points even when the disclosure is negative.

Sample size

We used all participants in the TwinsUK study with relevant phenotypic and genotypic data available. TwinsUK is large epidemiological study at King's College London with information on a range of cognitive and lifestyle variables as well as genetic and other omics data.

Data exclusions

All data exclusions are described in details in the methods section. In brief:
-For the epidemiological study, participants were excluded if they scored below 27 on the Mental State Examination (MMSE) to ensure cognitive health, if they underwent the The Paired Associates Learning (PAL) less than a year following onset of a disability and if they had a disability of unknown duration. Participants were also excluded if they failed genotyping QC as described in Methods: Genetic data.
-For the serum samples, participants were excluded if they scored below 27 on the Mental State Examination (MMSE) to ensure cognitive health and if they had neurological or psychiatric diseases or illnesses.
-For cellular data: no data were excluded.

Replication

There was no replication for the epidemiological study.
For the cellular work: Three biological replicates, each calculated by averaging 3 technical replicates.

Randomization

There was no randomisation for the epidemiological study. All individuals with information on cognitive variables, relevant covariates and genotype data were used and as the outcomes were continuous there were no experimental groups. All analyses were controlled for relevant covariates.
For the cellular work, randomisation does not apply as this is a cell line and the starting conditions before treatments are identical.

Blinding

Blinding was not relevant to our study. Both data collection and analyses required an understanding of the nature of the sample being collected/analyzed.

Reporting for specific materials, systems and methods

We require information from authors about some types of materials, experimental systems and methods used in many studies. Here, indicate whether each material, system or method listed is relevant to your study. If you are not sure if a list item applies to your research, read the appropriate section before selecting a response.

Materials & experimental systems

n/a	Involved in the study	
☐	☒	Antibodies
☐	☒	Eukaryotic cell lines
☒	☐	Palaeontology
☒	☐	Animals and other organisms
☐	☒	Human research participants
☒	☐	Clinical data

Methods

n/a	Involved in the study	
☒	☐	ChIP-seq
☒	☐	Flow cytometry
☒	☐	MRI-based neuroimaging

Antibodies

Antibodies used

Rabbit anti-Ki67 Abcam Ab15580
Mouse anti-ki67 CellSignalling 9449
Rabbit anti-CC3 CellSignalling 9664
Rabbit anti-Sox2 Abcam Ab5603
Mouse anti- Nestin AMD Millipore Mab5326
Mouse anti-H2a.X EMD Millipore 05-636-I
Rabbit anti-DCX Abcam Ab11267
Mouse anti-Map2 Abcam Ab11267
Rabbit anti-NRF2 Abcam Ab31163
555 Donkey Anti-rabbit IgG Life Technologies A-31572
488 Donkey Anti-mouse IgG Life Technologies A- 21202

Validation

Those antibodies are commonly used antibodies in the filed and have been validated by the companies. Refer to their website.

Eukaryotic cell lines

Policy information about cell lines

Cell line source(s)	The human hippocampal progenitor cells (HPC) cell line HPC0A07/03A were obtained from ReNeuron (www.reneuron.com/) and cultured as previously described in (Anacker et al., 2011) and (Johansson et al., 2008). The cells were obtained from 12-week-old foetal female tissue in accordance with UK and USA ethical and legal guidelines and transfected with the c-mycERTAM gene construct creating an immortalised cell line which proliferates in the presence the synthetic drug 4-hydroxy-tamoxifen (4-OHT) and spontaneously differentiates in its absence.
Authentication	No particular procedure other than Karyotyping and multiple gene expression analyses across parallel studies in the labs using this cell line.
Mycoplasma contamination	The cell line is routinely tested for mycoplasma and we can confirm it has never been tested positive.
Commonly misidentified lines (See ICLAC register)	*Name any commonly misidentified cell lines used in the study and provide a rationale for their use.*

Human research participants

Policy information about studies involving human research participants

Population characteristics	TwinsUK is made up of predominantly female (83%) white participants of European background. The registry now contains 51% monozygotic (MZ) and 49% dizygotic (DZ) twins aged 18–103 years, with information on a range of cognitive and lifestyle variables as well as genetic and other omics data. A full description can be consulted here: "Cohort Profile: TwinsUK and Healthy Ageing Twin Study" Int J Epidemiol. 2013 Feb; 42(1): 76–85. doi: 10.1093/ije/dyr207<br>This this particular article, sample size and demographic information for each trait is summarised in the (Figure S1).
Recruitment	The UK Adult Twin Registry (or TwinsUK Registry) is a cohort of volunteer adult twins from all over the United Kingdom. Volunteers were recruited through a series of media campaigns asking for volunteers willing to participate in research investigating common diseases. The Registry was started in 1992 with the primary aim of assessment of heritability of osteoarthritis and osteoporosis in women. The success of early studies led to rapid evolution of the registry and it now incorporates about 12 000 twins, both male and female aged 18–103 years, studied for a whole range of clinical and behavioural traits. The fact that its was started for women focused studies means there is a predominance of female participants in the cohort. See details here "Cohort Profile: TwinsUK and Healthy Ageing Twin Study" Int J Epidemiol. 2013 Feb; 42(1): 76–85. doi: 10.1093/ije/dyr207
Ethics oversight	Access to phenotypic and genotypic data of the TwinsUK cohort was requested and approved through the TwinsUK Resource Executive Committee (TREC) based at the Department of Twins Research and Genetic Epidemiology at Kings College London. Data from 2153 individuals with available cognitive data was received. TwinsUK procedures for sample and data collections have been described previously (Moayyeri et al., 2013; Steves et al., 2013) and were approved by the Guy's and St Thomas' Ethics Committee. TwinsUK data was collected in accordance with the declaration of Helsinki (1991).

Note that full information on the approval of the study protocol must also be provided in the manuscript.
